# Progesterone Attenuates SIRT1-Deficiency-Mediated Pre-Eclampsia

**DOI:** 10.3390/biom12030422

**Published:** 2022-03-09

**Authors:** Jiangnan Pei, Zhenzhen Liu, Chengjie Wang, Nan Chu, Lei Liu, Yao Tang, Haiyan Liu, Qianqian Xiang, Haidong Cheng, Mingqing Li, Weirong Gu

**Affiliations:** 1 Department of Obstetrics, Obstetrics and Gynecology Hospital of Fudan University, Shanghai 200011, China; 19111250017@fudan.edu.cn (J.P.); 21211250010@m.fudan.edu.cn (Z.L.); wangcj18@fudan.edu.cn (C.W.); 16111250001@fudan.edu.cn (N.C.); liulei11272@126.com (L.L.); tangtang061114158@163.com (Y.T.); haiyanliu11@fudan.edu.cn (H.L.); 2 Department of Obstetrics and Gynecology, Peking University Third Hospital, Beijing 100191, China; chestnut1013@163.com

**Keywords:** progesterone, pre-eclampsia, SIRT1, pre-eclampsia-like mice, SRT2104, metformin

## Abstract

Pre-eclampsia is a severe hypertensive disorder of pregnancy (HDP), mainly characterized by new-onset hypertension with proteinuria after 20-week gestation. Sirtuin1 (SIRT1), a class III histone deacetylase, is associated with the regulation of various pathophysiological processes, including inflammation, immune response, metabolism, and autophagy. However, the effect of SIRT1 in the pathogenesis of pre-eclampsia remains to be elucidated. In this study, we found that the expression of SIRT1 was relatively lower in the placentas and serum samples of pre-eclampsia patients. Typical pre-eclampsia-like symptoms, such as hypertension, proteinuria, fetal growth restriction, kidney injury, and a narrow placental labyrinth layer, were observed in SIRT1 knockdown (SIRT1^+/−^) mice. Of note, these performances could be improved after the intraperitoneal injection of SIRT1 agonist SRT2104. More importantly, we found that the efficacy of progesterone on attenuating symptoms of PE was profoundly better than that of metformin in SIRT1^+/−^ mice. In addition, our results suggested that progesterone can promote the invasion and inhibit the apoptosis of trophoblasts. These data suggest that SIRT1 plays an important role in pre-eclampsia and that progesterone alleviates pre-eclampsia-like symptoms mediated by SIRT1 deficiency.

## 1. Introduction

Pre-eclampsia (PE) is defined as new-onset hypertension with proteinuria after 20-week gestation. In severe cases, it can also combine with renal, cardiac, pulmonary, hepatic, and neurological dysfunction; hematologic disturbances; fetal growth restriction; stillbirth; and even maternal death [1]. Pre-eclampsia impacts 2 to 8% of all pregnancies worldwide, which is a major cause of maternal and fetal morbidity and mortality, and the only known cure for this complication is delivery [2,3]. Elucidating the pathophysiological mechanisms of pre-eclampsia will help us accurately prevent, manage, and treat it to avoid maternal and fetal losses.

SIRT1 (Sirtuin 1) is an NAD+-dependent deacetylase that plays critical roles in many biological events, including metabolism, immune response, cellular inflammation, autophagy, and senescence [4,5,6,7,8,9]. Furthermore, it is reported that SIRT1 can regulate cell migration and invasion in several cancer cells by deacetylating various substrates [10,11,12], and the vascular remodeling of the placenta also depends on the invasive properties of trophoblasts, so we consider whether SIRT1 also affects pre-eclampsia. Additionally, as an important factor causing aging, SIRT1 deficiency can lead to placental senescence [13]. However, whether SIRT1 directly induces pre-eclampsia as well as the comprehensive molecular mechanisms between SIRT1 and pre-eclampsia are still poorly understood.

A few studies have suggested that SIRT1 may contribute to progesterone (P4) resistance. The expression of P4 target genes was decreased in SIRT1-overexpression mice [14,15]. P4 is a cholesterol-derived hormone critical for establishing and maintaining pregnancy [16]. It is reported that circulating P4 is significantly lower in PE women than controls, and P4 attenuates hypertension in response to placental ischemia in reduced uterine perfusion pressure (RUPP) rats [17]. P4 is also crucial for decidualization and blocks inflammation in the endometrium [18]. However, whether P4 improves PE symptoms through the SIRT1 pathway is still not clear.

In our study, we constructed a pre-eclampsia-like mouse model by knocking down SIRT1, clarifying the important role of SIRT1 in the progression of pre-eclampsia, providing new ideas for exploring the pathogenesis of pre-eclampsia, and the discovery of the therapeutic effect of P4 on pre-eclampsia may provide a theoretical basis for clinical prevention and treatment of pre-eclampsia.

## 2. Materials and Methods

### 2.1. Human Samples

This research was approved by the local ethics committees of Obstetrics and Gynecology Hospital of Fudan University (Shanghai, China) and Peking University Third Hospital (Beijing, China). We collected 95 normal pregnancy women (NP) and 76 pre-eclampsia women (PE). PE patients were obtained according to the criteria of the American College of Obstetricians and Gynecologists [19]. Table 1 shows the detailed clinical characteristics of the 171 volunteers. All those volunteers signed informed consent. Serum samples collected from 95 normal pregnant women and 76 PE patients were used to detect the SIRT1 expression level using ELISA Kit (ab171573, Abcam, Cambridge, UK). Placentas were cut off from the maternal side for immunohistochemistry (IHC) tests and Western blot (WB).

### 2.2. Animal Experiments

#### 2.2.1. Establishment of the Mouse Model

All animals were housed in a temperature-controlled room (23 °C) with a 12:12 light: dark cycle.

SIRT1 knockdown mice (SIRT1^+/−^) (HE) were generated by crossbreeding SIRT1*^flox/flox^* (WT) mice with broad-expression Cre transgenic mice. The Cre transgenic mice were provided by the Shanghai Model Organisms Center (Shanghai, China), while the SIRT1*^flox/flox^* mice were purchased from the Jackson Lab (029603, the Jackson Laboratory, Farmington, CT, USA). Successful knockdown of SIRT1 in mice was confirmed with PCR and immunofluorescence. 

The PCR cycling conditions for SIRT1 were a primary denaturation at 94 °C for 3 min, followed by 35 cycles of 30 s at 94 °C, annealing temperature at 60 °C for 30 s, and 72 °C for 60 s, with a final extension of 5 min at 72 °C. Primer sequences for PCR were as follows: Cre: forward AGGCGGATTTCTGAGTTCGA and reverse CGTCCCTTGTAATGTTTCCC and floxed SIRT1 gene: forward AGGAATCCCACAGGAGACAG and reverse GGTTAAGATTAGCCCATTAAAGC.

SIRT1^+/−^ female mice at 8 weeks of age were individually mated with SIRT1^+/−^ male mice. The initiation of pregnancy was marked by the presence of postcoital vaginal plug at gestation day (GD) 0.5 for early pregnancy study.

#### 2.2.2. Systolic Blood Pressure (SBP) Measurement

The systolic blood pressure of the pregnant mice in each group was measured using a non-invasive blood pressure monitor with a volume pressure-recording sensor and an occlusion tail cuff (BP-2000 Series II, Visitech Systems, Apex, NC, USA). We monitored the systolic blood pressure of mice for 3 consecutive days before officially starting the experiments to allow them to adapt to the environment. Then, SBP was monitored every other day from GD2 to GD17, whether it was the experimental groups or the control groups. It is important to note that SBP should be measured before administration to ensure that the value of SBP is not disturbed.

#### 2.2.3. Animal Grouping and Treatment

SRT2104 is a highly selective small-molecule activator of SIRT1 [20], and was demonstrated to be safe in animals and humans [21,22,23]. Studies showed that SRT2104 could significantly elevate SIRT1 protein level in C57BL/6 mice without altering SIRT1 mRNA (100 mg/kg/day, intragastric administration for 24 weeks) [24,25].

Before officially starting the experiment, we first explored the side effects of SRT2104 on mice. We divided 15 SIRT1^+/−^ mice into four groups, intragastric administration with PBS (*n* = 3), SRT2104 (50 mg/kg) (*n* = 5), SRT2104 (100 mg/kg) (*n* = 4), and SRT2104 (200 mg/kg) (*n* = 3), respectively, every other day from GD2 to GD17, then detected the level of the concentration of ALT, AST, BUN, and Cr in mice serum at GD18 by Simuwubio (Simuwubio, Shanghai, China).

Then, the remaining 27 SIRT1^+/−^ mice were randomly divided into five groups for intraperitoneal injection of vehicle (*n* = 8), SRT2104 (25 mg/kg) (*n* = 8), metformin (100 mg/kg) (*n* = 3), metformin (200 mg/kg) (*n* = 5), and P4 (3 mg/kg) (*n* = 3). SRT2104 (HY-15262, MedChemExpress, Monmouth Junction, NJ, USA) was dissolved in vehicle (5% DMSO, 40% PEG300, 5% Tween80, 50% saline) and intraperitoneally injected every other day from GD2 to GD17. Metformin (HY-B0627, MedChemExpress, USA) was dissolved in vehicle and intraperitoneally injected at GD7.5, GD10.5, GD13.5, GD16.5. The injection dose of metformin was chosen based on previous reports [26,27]. P4 (P0130, Sigma, St. Louis, MO, USA) was dissolved in vehicle and intraperitoneally injected every day from GD7.5 to GD18. The injection dose of P4 was chosen based on a previous report [16].

#### 2.2.4. Mouse Urine Collection and Detection

The 24-h urine of the pregnancy mice in both groups was collected during the 17–18th days of gestation. The concentration of urinary protein was detected using Albuwell M Test Kit (1011, Exocell, Logan Township, NJ, USA), following the protocol.

#### 2.2.5. Placentas, Fetuses, and Kidneys Collection

Mice were euthanized using isoflurane, and their placentas, fetuses, and kidneys were collected on the 18th day of gestation. The placentas and fetuses were weighed and imaged. The kidneys and placental tissues were fixed in 4% paraformaldehyde overnight, embedded in paraffin, and then cut into 5 μm sections for subsequent experiments.

### 2.3. Western Blot Analysis

Total protein was isolated from placental tissues of 7 normal pregnancy women (NP) and 7 women with pre-eclampsia with severe features (sPE). The diagnostic criteria for pre-eclampsia with severe features refer to American College of Obstetricians and Gynecologists’ Practice Bulletin on Gestational Hypertension and Pre-eclampsia [28]. The protein samples were separated by electrophoresis, transferred to polyvinylidene difluoride (PVDF) membrane, and then incubated with the following primary antibodies anti-SIRT1 (dilution 1:5000, ab32441, Abcam, UK) overnight at 4 °C. On second day, the PVDF membrane was incubated with horseradish peroxidase (HRP)-conjugated secondary antibody (dilution 1:1000, 7074 S, Cell Signaling Technology, USA) for 2 h at room temperature. GAPDH (1:5000, ab181602, Abcam, UK) was used as an internal standard. The signals were visualized with an ECL solution (P0018FS, Beyotime, Shanghai, China). Images were obtained using Amersham Imager 600 (GE Healthcare, Waukesha, WI, USA).

### 2.4. Immunohistochemistry (IHC) and Immunofluorescence (IF)

The human placental sections were deparaffinized and treated with 3% H_2_O_2_ to block the endogenous peroxidase activity. Then, the sections were pretreated heat-mediated antigen retrieval using sodium citrate buffer or Tris/EDTA buffer and incubated with primary antibodies against SIRT1 (dilution 1:100, ab32441, Abcam, UK) at 4 °C overnight. Positive signals were visualized by incubation with HRP-conjugated secondary antibodies using microscope (Olympus IX73).

The mice placental sections were used for immunofluorescence, and the process is similar to immunohistochemistry. The sections were incubated with primary antibodies against CK7 (dilution 1:5000, ab181598, Abcam), SIRT1 (dilution 1:100, ab32441, Abcam) at 4 °C overnight. Positive signals were visualized by incubation with fluorophore-conjugated secondary antibodies. The slides were mounted with mounting medium containing DAPI (Abcam) and then viewed and imaged under a fluorescence microscope (Olympus IX73).

### 2.5. Masson Staining and PAS Staining

The Masson staining kit (ab150669, Abcam) and PAS staining kit (ab150680, Abcam) were used for histological analysis of the mice kidney sections. After dehydration with ethyl alcohol and blocking with neutral gum, we used light microscope (Olympus IX73) to observe and evaluate the kidney injury. For Masson staining, the glomerulus is bloodless with pre-eclampsia; for PAS, the capillary lumens are occluded by swollen endothelial cells in typical pre-eclampsia kidney pathology.

The mice placental sections were stained with Masson staining kit (ab150669, Abcam) and imaged under the light microscope (Olympus IX73). Then, we used ImageJ software to measure the area of the labyrinth layer and junctional zone of placentas. The ratio between the labyrinth layer and junctional zone is considered an indicator of placental function [29].

### 2.6. Cell Experiment

#### 2.6.1. Cell Culture

The trophoblastic HTR-8/SVneo cells (CRL-3271, Manassas, VA, ATCC, USA) were cultured in DMEM/F12 medium with 10% fetal bovine serum (FBS) (Gibico, Pittsburgh, PA, USA) under standard culturing conditions (37 °C and 5% CO_2_ in humidified atmosphere).

#### 2.6.2. Lentivirus Transfection

Lentiviruses carrying short hairpin RNA (shRNA) targeting human Sirtuin1 (SIRT1) lentivirus vectors (GV248) were purchased from GeneChem (Shanghai, China). HTR8/SVneo cells were transfected with viruses (multiplicity of infection = 20) for 24 h according to the manufacturer’s instructions. Then, the cells were transferred to fresh medium containing puromycin (1 μg/mL) for the selection of stable clones after three passages. The shRNA sequence was 5′-GGCTTGATGGTAATCAGTA-3′. Then, the cells were divided into two groups, control and SIRT1*KO* groups, for P4 treatment.

#### 2.6.3. Cell Treatment

The HTR-8/SVneo cells were divided into 4 groups: P4, Met, SRT2104, and vehicle groups, respectively treated with P4 (0, 10^−8^, 10^−7^, 10^−6^, 10^−5^, 10^−4^ mol/L; Sigma), metformin (5 mM, MedChem Express), SRT2104 (1 uM, MedChem Express), and 0.1% DMSO for 24 h. The concentration gradient of P4 was chosen based on the reference [30].

#### 2.6.4. Cell Proliferation Assay

Cell counting kit-8 (CCK-8) (YEASEN, Beijing, China) was used to test the cell proliferation ability according to the manufacturer’s instructions. HTR-8/SVneo cells (96-well plates, 1 × 10^4^ cells/well) from P4 and vehicle groups were cultured with 10 μL/well CCK-8 solution for 1.5 h. The absorbance was measured at 450 nm using a microplate reader (Bio-Rad, Hercules, CA, USA).

#### 2.6.5. Transwell Assay

Matrigel (dilution 1:8, BD Biosciences, San Diego, CA, USA) was added to the upper chamber of Transwell chambers, and the Transwell chambers were placed in a 24-well plate and incubated overnight at 4 °C. Then, 200 μL (HTR-8/SVneo, 1 × 10^5^ cells/well) DMEM/F12 suspension with or without 10% FBS was added to the upper chamber, and 600 μL DMEM/F12 containing 10% FBS was added to the lower chamber. The cells were cultured for 48 h at 37 °C in a 5% CO_2_ incubator. The 24-well plate was removed, and the upper chamber medium and nonpenetrating cells were gently wiped off with a cotton swab, washed three times with phosphate-buffered saline (PBS), fixed with 4% paraformaldehyde for 30 min, and stained with crystal violet for 20 min. Subsequently, random photographs were acquired under an inverted microscope (×200), and 5 visual fields were counted in each chamber. The number of invaded cells was counted using Image J software (National Institutes of Health, Bethesda, MD, USA).

#### 2.6.6. Apoptosis Assay

HTR-8/SVneo cells from P4 and vehicle groups were stained with Annexin V and 7-AAD (ab214663, Abcam, UK) for 15 min at RT in the dark. Fluorescence intensities were analyzed by flow cytometry by using the BD Cell Quest Pro software.

### 2.7. Statistical Analysis

All results were analyzed using GraphPad Prism9.2.0 (GraphPad Software, San Diego, CA, USA); Friedman’s test was used to analyze the variance. Differences between groups were analyzed by one-way ANOVA (and nonparametric or mixed) and unpaired Student’s test (and nonparametric tests), results were presented as the mean ± standard deviation (SD), and values with *p* < 0.05 were supposed to be statistically significant.

## 3. Results

### 3.1. SIRT1 Was Significantly Lower in the Placentas and Serum Samples of Pre-Eclampsia Patients

**Figure 1:**
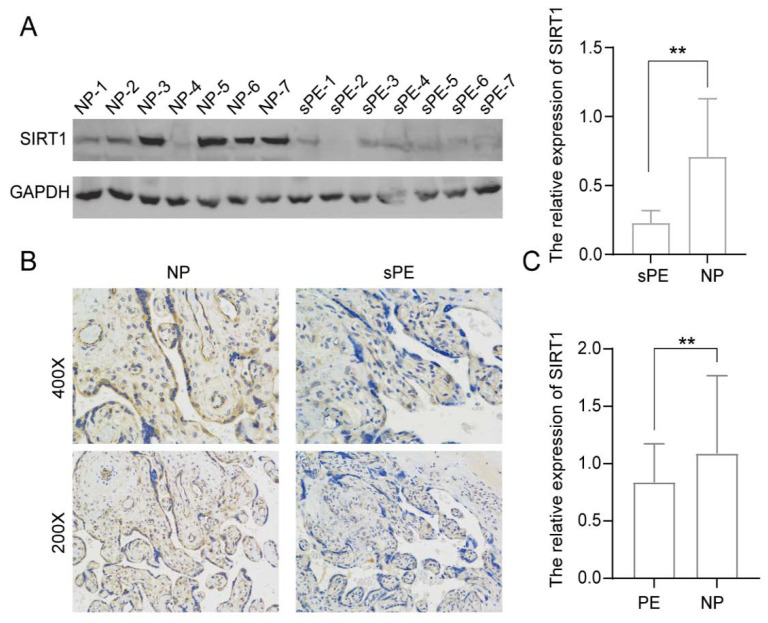
SIRT1 was significantly lower in the placentas and serum samples of pre-eclampsia patients. (**A**) Western blot analysis of SIRT1 protein levels in placental tissues from normal pregnancy women (NP, *n* = 7) and women with pre-eclampsia with severe features (sPE, *n* = 7). (**B**) Immunohistochemistry for SIRT1 in placental tissues from NP women (*n* = 7) and sPE women (*n* = 7). (**C**) ELISA analysis of the SIRT1 concentration in the serum of PE women (*n* = 76) and gestational age-matched NP women (*n* = 95). Error bars, mean ± SD. The data were analyzed by an unpaired Student’s test. ** *p* < 0.01.

To study the role of SIRT1 in PE, we evaluated the level of SIRT1 expression in human placental tissues from women with pre-eclampsia with severe features (sPE) (*n* = 7) and normal pregnant women (NP) (*n* = 7) (Figure 1). Compared with controls, the level of SIRT1 expression was significantly lower in the placentas of sPE patients (Figure 1A), as detected by Western Blotting. Immunohistochemistry results also showed that SIRT1 expression was apparently decreased in the placentas of sPE patients (Figure 1B).

Next, the levels of SIRT1 in serum samples from 76 PE patients and 95 NP women were detected by ELISA. The result indicated that the SIRT1 expression from serum samples was significantly decreased in the PE patients (Figure 1C, PE vs. NP: 0.8379 ± 0.3370 vs. 1.089 ± 0.6779 relative absorbance).

### 3.2. SIRT1^+/−^ Mice Demonstrated Pre-Eclampsia-like Symptoms

**Figure 2:**
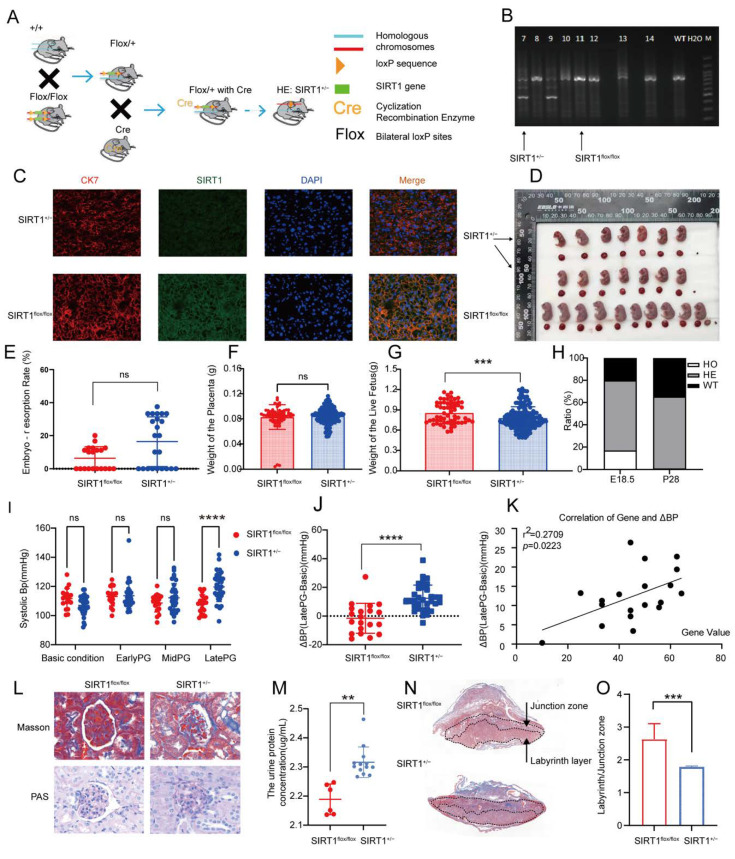
SIRT1^+/−^ mice demonstrated pre-eclampsia-like symptoms. (**A**) Schematic diagram of SIRT1^+/−^ mice construction. (**B**) Genotype of SIRT1^+/−^ mice using PCR and agarose gel electrophoresis. (**C**) Immuofluorescence for SIRT1 in mice placental tissues from SIRT1^flox/flox^ (*n* = 3) and SIRT1^+/−^ (*n* = 7) mice. (**D**) Representative appearance of fetuses and placentas from SIRT1^flox/flox^ and SIRT1^+/−^ groups. (**E**–**G**) The embryo-resorption rate (**E**), the weight of the placenta (**F**), and the weight of the live fetus (**G**) in SIRT1^flox/flox^ (*n* = 19) and SIRT1^+/−^ (*n* = 31) mice. (**H**) The gene ratio of fetuses at E18.5 and P28 (E18.5: the 18.5th day of gestation, P28: the 28th day in postnatal age). (**I**) The systolic blood pressure (SBP) at basic condition, early PG (pregnancy), mid PG, late PG from SIRT1^flox/flox^ (*n* = 20), and SIRT1^+/−^ (*n* = 33) groups. (**J**) ΔBP (late PG—basic condition) in SIRT1^flox/flox^ (*n* = 20) and SIRT1^+/−^ (*n* = 33) groups. (**K**) The correlation of ΔBP and SIRT1 gene values in fetuses. (**L**) Masson staining and PAS staining of mice kidney tissues from SIRT1^flox/flox^ (*n* = 3) and SIRT1^+/−^ (*n* = 7) groups. (**M**) The concentration of urinary protein in SIRT1^flox/flox^ (*n* = 6) and SIRT1^+/−^ (*n* = 13) groups at late PG. (**N**) Masson staining of mice placental tissues in SIRT1^flox/flox^ (*n* = 3) and SIRT1^+/−^ (*n* = 7) groups. (**O**) The labyrinth/junctional zone ratio of each group. Error bars, mean ± SD. The data were analyzed by one-way ANOVA (and nonparametric or mixed) and unpaired Student’s test (and nonparametric tests). ** *p* < 0.01; *** *p* < 0.001; **** *p* < 0.0001, ns: no significance.

To investigate the impact of SIRT1 on pre-eclampsia, we developed a SIRT1 knockdown (SIRT1^+/−^) mouse model from SIRT1*^flox/flox^* mice, and the gene knockdown and mice mating strategies are shown in Figure 2A. We found that all SIRT1^−/−^ mice (HO) died at postnatal 28 days (Figure 2H), which was consistent with previous research that SIRT1^−/−^ mice suffered from severe growth retardation and developmental defects [29,31,32,33]. Therefore, we selected the SIRT1^+/−^ mice to finally conduct our research. To confirm that the construction of the SIRT1^+/−^ mice model was successful, we detected the genotype of the model mice by PCR and agarose gel electrophoresis (Figure 2B), and the mice with SIRT1^+/−^ were selected for the following treatment. The expression of SIRT1 in placental tissues of SIRT1^+/−^ mice was downregulated compared with SIRT1*^flox/flox^* mice by immunofluorescence, as shown in Figure 2C.

Subsequently, to verify whether the model mice showed pre-eclampsia-like symptoms, we conducted a series of observations and experiments. We found that the embryo-resorption rate was almost normal in SIRT1^+/−^ mice (Figure 2E). Additionally, there was no significant difference in placental weight among the two groups (Figure 2F). Since PE can cause fetal growth restriction (FGR), we analyzed the weight of the live fetus in SIRT1*^flox/flox^* and SIRT1^+/−^ groups, showing that the weight of the live fetus was dramatically lower in SIRT1^+/−^ mice (Figure 2G, SIRT1^+/−^ vs. WT: 0.7803 ± 0.1651 vs. 0.8559 ± 0.1585 g). Representative images of fetuses and placentas from each group are shown in Figure 2D, and the size of the fetus was smaller compared with WT groups. Subsequently, we analyzed the changes in systolic blood pressure (SBP). Interestingly, the level of SBP increased dramatically at late pregnancy in SIRT1^+/−^ mice (Figure 2I, SIRT1^+/−^ vs. WT: 119.6 ± 9.952 vs. 108 ± 6.340 mmHg). To understand the level of elevated BP, we calculated the difference of SBP between late pregnancy and the basic condition. We found that the ΔBP presented an increasingly high level in SIRT1^+/−^ groups (Figure 2J, SIRT1^+/−^ vs. WT: 12.45 ± 9.186 vs. −1.562 ± 10.47 mmHg). Intriguingly, the level of ΔBP had a positive correlation with the SIRT1 gene value in fetuses (Figure 2K).

In addition, we detected the concentration of urinary protein and investigated the renal pathological changes of each group. The concentration of urinary protein was significantly increased in late-pregnancy SIRT1^+/−^ mice (Figure 2M, SIRT1^+/−^ vs. WT: 2.316 ± 0.05245 vs. 2.189 ± 0.05252 ug/mL). Furthermore, SIRT1^+/−^ mice showed typical pre-eclampsia-related glomerular injuries, compared to the SIRT1*^flox/flox^* group (Figure 2L).

To explore the pathological changes in the placenta, we compared the labyrinth/junction zone ratio in the placentas of each group. The labyrinth layer is the functional layer of the placenta [32]. As a result, we observed that the labyrinth layer was narrow in SIRT1^+/−^ mice compared with SIRT1*^flox/flox^* (Figure 2N), and the labyrinth/junction zone ratio was significantly decreased in the SIRT1^+/−^ group compared with the SIRT1*^flox/flox^* group (Figure 2O).

### 3.3. SRT2104 Significantly Diminished the PE-like Symptoms in SIRT1^+/−^ Mice

**Figure 3:**
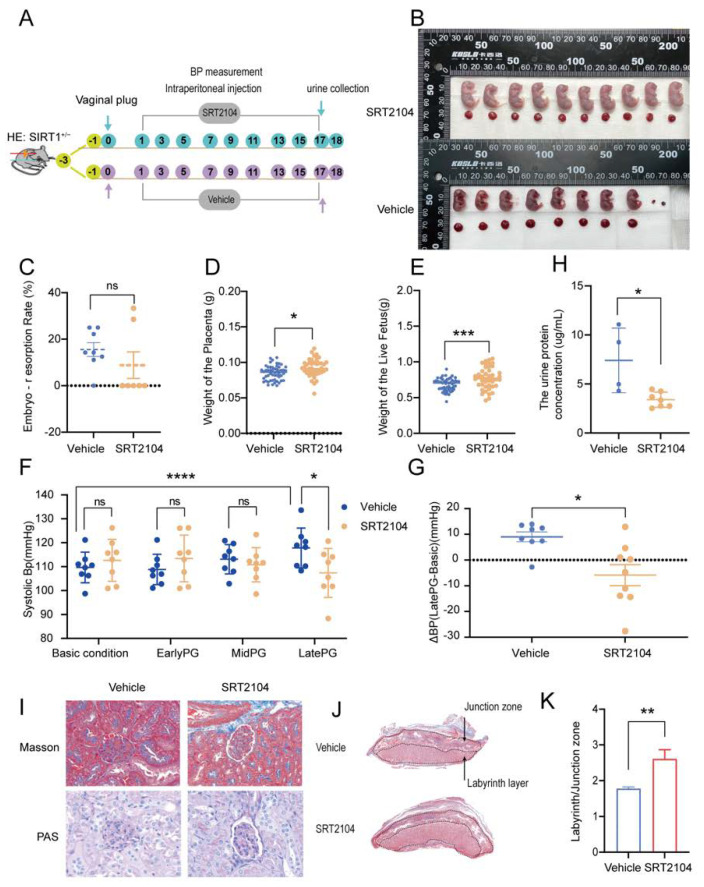
SRT2104 significantly improved the PE-like symptoms in SIRT1^+/−^ mice. (**A**) Schematic diagram of the treatment of SIRT1^+/−^ mice from early pregnancy to late pregnancy. (**B**) Representative appearance of fetuses and placentas from vehicle and SRT2104 groups. (**C**–**G**) The embryo-resorption rate (**C**), the weight of the placenta (**D**), and the weight of the live fetus (**E**) in vehicle (*n* = 8) and SRT2104 (*n* = 7) groups. (**F**) The SBP at basic condition, early PG, and mid PG, late PG from vehicle (*n* = 8) and SRT2104 (*n* = 9) groups. (**G**) ΔBP (late PG—basic condition) in vehicle (*n* = 8) and SRT2104 (*n* = 9) groups. (**H**) The concentration of urinary protein in vehicle (*n* = 4) and SRT2104 (*n* = 7) group at late PG. (**I**) Masson staining and PAS staining of mice kidney tissues from vehicle (*n* = 4) and SRT2104 (*n* = 4) groups. (**J**) Masson staining of mice placental tissues in vehicle (*n* = 4) and SRT2104 (*n* = 4) groups. (**K**) The labyrinth/junctional zone ratio of each group. Error bars, mean ± SD. The data were analyzed by one-way ANOVA (and nonparametric or mixed) and unpaired Student’s test (and nonparametric tests). * *p* < 0.05; ** *p* < 0.01; *** *p* < 0.001; **** *p* < 0.0001, ns: no significance.

To verify the function of SIRT1 in pre-eclampsia, we intraperitoneally injected the SIRT1^+/−^ mice with SIRT1 agonist SRT2104 every other day from GD2 to GD17. The process of treating mice is shown in Figure 3A. Before starting the formal experiments, it had been confirmed that the side effect of SRT2104 was mild (Figure S1A–D).

We found that SRT2104 could reverse the pre-eclampsia-like symptoms driven by SIRT1 knockdown. After treating SIRT1^+/−^ mice with SRT2104, the embryo-resorption rate had no significant change (Figure 3C), the weight of the placenta was increased slightly (Figure 3D, vehicle vs. SRT2104: 0.08519 ± 0.01009 vs. 0.09237 ± 0.01213 g), while the weight of the live fetus was dramatically increased (Figure 3E, vehicle vs. SRT2104: 0.6808 ± 0.08630 vs. 0.7719 ± 0.1483 g). These results were consistent with the representative images of fetuses and placentas (Figure 3B). The level of systolic blood pressure at late pregnancy (Figure 3F, vehicle vs. SRT2104: 117.8 ± 8.311 vs. 107.4 ± 10.21 mmHg) and the ΔBP (Figure 3G, vehicle vs. SRT2104: 8.158 ± 9.212 vs. −5.234 ± 12.86 mmHg) were both decreased compared with the vehicle group. The kidney injury was improved with SRT2104, presenting a decreased urinary protein concentration in late pregnancy (Figure 3H, vehicle vs. SRT2104, 7.397 ± 3.293 vs. 3.392 ± 0.7711 ug/mL) and almost normal glomerulus morphology (Figure 3I). The placental selectins also showed a widened labyrinth layer after using SRT2104 for intraperitoneal injection (Figure 3J,K).

However, the effect was not very apparent when we treated SIRT1^+/−^ mice with SRT2104 via intragastric administration. The embryo-resorption rate had no visible change (Figure S1E). The weight of the placenta and fetus was significantly increased (Figure S1F,G) when the dose of SRT2104 was 50 mg. Additionally, there was also an increase in the weight of fetus when the dose was 100 mg (Figure S1G). However, we did not see any difference in the level of systolic blood pressure (Figure S1H).

### 3.4. P4 Could Alleviate Hypertension in SIRT1^+/−^ Mice and Promote the Invasion and Inhibit the Apoptosis of Trophoblasts

**Figure 4:**
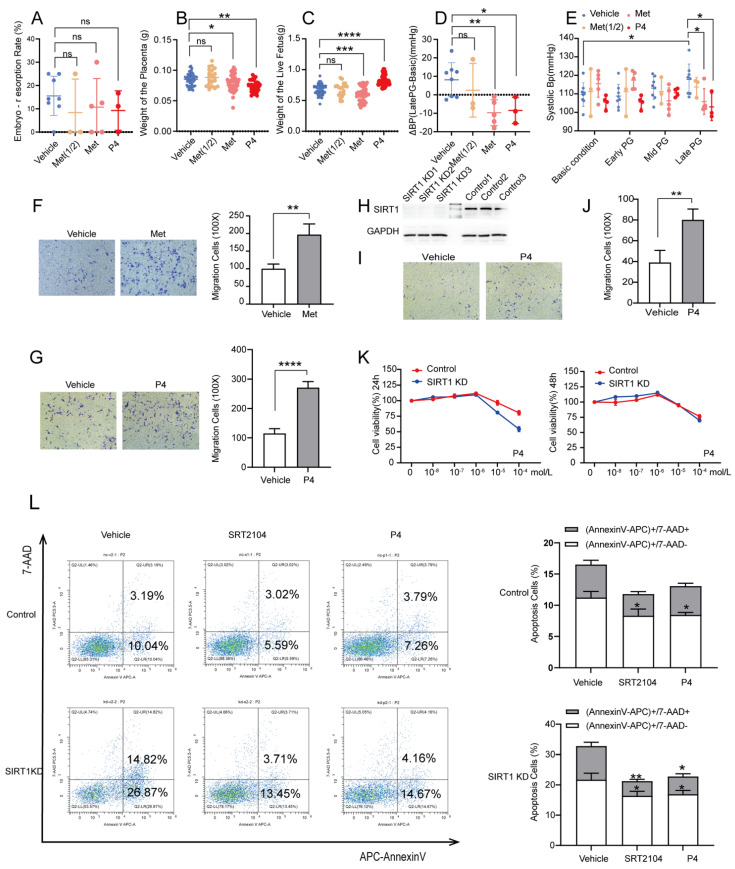
P4 could alleviate hypertension in SIRT1^+/−^ mice and promote the invasion and inhibit the apoptosis of trophoblasts. (**A**–**E**) The embryo-resorption rate (**A**), the weight of the placenta (**B**), and the weight of the live fetus (**C**) in vehicle (*n* = 8), Met (1/2) (*n* = 3), Met (*n* = 5), and P4 (*n* = 3) groups. (**D**) ΔBP (late PG—basic condition) in vehicle (*n* = 8), Met (1/2) (*n* = 3), Met (*n* = 5), and P4 (*n* = 3) groups. (**E**) The SBP at basic condition, early PG, mid PG, late PG from vehicle (*n* = 8), Met (1/2) (*n* = 3), Met (*n* = 5), and P4 (*n* = 3) groups. (**F**,**G**) The invasion ability of Transwell assay in vehicle, Met (**F**), and P4 (**G**) groups of control group. (**H**) Western blot analysis of SIRT1 protein levels in trophoblasts of SIRT1 *KD* and control groups. (**I**) The invasion ability of Transwell assay of vehicle and P4 groups in SIRT1 *KD* trophoblasts. (**J**) Statistics of migrating cells. (**K**) The proliferation function checked by CCK8 at 24 h and 48 h treated with P4 (concentration: 0, 10^−8^, 10^−7^, 10^−6^, 10^−5^, 10^−4^ mol/L) in SIRT1 *KD* trophoblasts. (**L**) The apoptosis assay of vehicle, SRT2104 and P4 groups in SIRT1 *KD* trophoblasts. Error bars, mean ± SD. The data were analyzed by one-way ANOVA (and nonparametric or mixed) and unpaired Student’s test (and nonparametric tests). * *p* < 0.05; ** *p* < 0.01; *** *p* < 0.001; **** *p* < 0.0001, ns: no significance.

Previous research reported that P4 and metformin could improve the symptoms of pre-eclampsia [16,31], but the results are still controversial. To determine whether P4 and metformin could be effective drugs for pre-eclampsia, we treated pregnant SIRT1^+/−^ mice with P4 and metformin. We found that there was no significant difference in the embryo-resorption rate (Figure 4A), and the weight of the placenta was decreased in both groups (Figure 4B), which may be due to the batch difference caused by the residual decidua in part of the placentae during the initial dissection. However, there was a big difference between the two groups in the weight of the live fetus. P4 could elevate the weight of the live fetus, while the metformin decreased the fetal weight (Figure 4C, vehicle vs. P4, 0.6808 ± 0.08630 vs. 0.6037 ± 0.1168 g; vehicle vs. metformin, 0.6808 ± 0.08630 vs. 0.8290 ± 0.08013 g). In addition, we found that the increased systolic blood pressure at late gestation was notably attenuated after administration of P4 or metformin (Figure 4E, vehicle vs. P4, 117.8 ± 8.311 vs. 103.0 ± 7.388 mmHg; vehicle vs. metformin, 117.8 ± 8.311 vs. 105.8 ± 7.001 mmHg). Additionally, ΔBP also presented a downward tendency whether in Met groups and P4 groups (Figure 4D, vehicle vs. P4, 8.158 ± 9.212 vs. −8.477 ± 7.138 mmHg; vehicle vs. metformin, 8.158 ± 9.212 vs. −9.680 ± 7.041 mmHg). The above were all in the case of sufficient metformin. In the case of half the amount of metformin, there was almost no effect (Figure 4A–E). Furthermore, we treated HTR8/SVneo cells with P4 and metformin; the results showed that trophoblast invasion was elevated both in Met and P4 groups, and we saw a greater increase in P4 groups (Figure 4F,G). Therefore, we believed that P4 had better efficacy in diminishing the symptoms of pre-eclampsia.

To further verify the mechanisms of P4 in pre-eclampsia, we knocked down SIRT1 in HTR8/SVneo with lentivirus. The expression of SIRT1 was significantly decreased after transfection (Figure 4H). The trophoblast invasion was significantly decreased after SIRT1 knockdown, and P4 could reverse this result (Figure 4I,J). Additionally, there was no significant change in the proliferation of trophoblast when treated with low-dose P4, whether it was treated for 24 h or 48 h (Figure 4K). However, the apoptosis of trophoblast was dramatically decreased after treatment with P4, which is similar to SRT2104 (Figure 4L). These results demonstrated that P4 could enhance the invasion and inhibit the apoptosis of trophoblasts in order to prevent the progress of pre-eclampsia.

## 4. Discussion

Pre-eclampsia is a severe pregnancy-related complication with the onset of hypertension after week 20 of pregnancy. Pre-eclampsia is mainly characterized by inadequate trophoblasts invasion, which can be caused by autophagy, abnormal metabolism, inflammation, and oxidative stress [34,35,36]. However, the comprehensive mechanisms of PE remain unclear.

**Figure 5:**
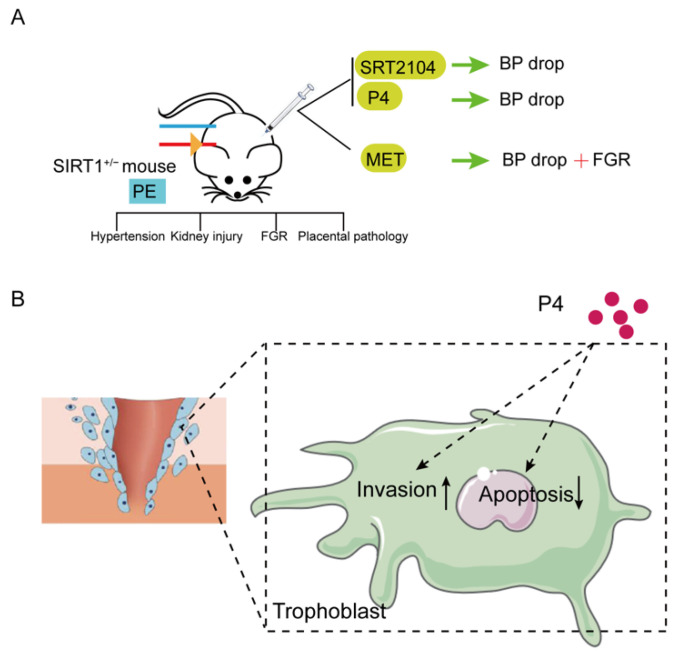
Summary diagram. (**A**)SIRT1 knockdown (SIRT1^+/−^) mice showed typical pre-eclampsia-like symptoms, such as hypertension, kidney injury, FGR, and placental pathology. These performances could be improved after intraperitoneal injection of SIRT1 agonist SRT2104 and P4. Metformin could also reduce the elevated blood pressure, but caused FGR. (**B**) Additionally, P4 could promote the invasion and inhibit the apoptosis of trophoblasts. (PE: pre-eclampsia; FGR: fetal growth restriction; P4: progesterone; Met: metformin.).

In our research, we found that the expression of SIRT1 was significantly lower in placentas and serum samples of pre-eclampsia patients. SIRT1 knockdown (SIRT1^+/−^) mice showed typical pre-eclampsia-like symptoms, such as hypertension, proteinuria, fetal growth restriction, kidney injury, and a narrowed placental labyrinth layer, implying that the construction of the PE-like mice model was successful, and SIRT1 heterozygote knockout is sufficient to drive the symptoms of pre-eclampsia. Interestingly, SRT2104, P4, and metformin all significantly lower blood pressure in SIRT1^+/−^ mice, while metformin can also cause fetal growth restriction. In addition, our results suggested that P4 can promote the invasion and inhibit the apoptosis of trophoblasts (Figure 5).

SIRT1 might be a critical mediator in the pathogenesis of pre-eclampsia by multiple pathways. Previous research reported that alteration of autophagy activity in trophoblasts affected the invasion function of trophoblasts and was implicated in the pathophysiology of PE [37,38,39,40,41]. SIRT1 can deacetylate the LIR motif of LC3 to affect cell autophagy [9]. In addition, studies have shown that SIRT3 can regulate trophoblasts’ autophagy by activating the AMPK-mTOR pathway and reducing GPX4 levels [42]. SIRT1 can module the oxidative stress and apoptosis of trophoblasts by autophagy [34]. We speculated that SIRT1 might regulate trophoblasts’ autophagy activity by deacetylating a certain substrate, thereby leading to the occurrence of pre-eclampsia, but further experimental verification is needed. Furthermore, L-NAME (N-nitro-L-arginine methyl ester)-induced pre-eclampsia animal model is a common pre-eclampsia animal model, mainly by inhibiting nitric oxide (NO) synthase activity, as NO is an important vasodilator [43]. It has been reported that SIRT1 induced endothelial NO increase by directly deacetylating eNOs or by activating FOXO and AMPK and is thereby involved in the pathogenesis of pre-eclampsia [44,45,46,47]. However, since SIRT1 has a wide range of functions in vivo and may induce the occurrence of pre-eclampsia through multiple pathways, the comprehensive mechanisms remain unclear.

SRT2104 can eliminate pre-eclampsia-like symptoms induced by SIRT1 knockdown. SRT2104 is a selected SIRT1 agonist and can elevate the expression of SIRT1 [24]. Studies showed that SRT2104 has positive effects on cardiovascular disease and diabetic nephropathy [48,49], but no studies indicate that SRT2104 has the ability to treat pre-eclampsia. In our study, the pre-eclampsia-like manifestations of SIRT1^+/−^ mice were significantly improved after intraperitoneal injection of SRT2104. Therefore, we speculated that SRT2104 might correct the manifestations of pre-eclampsia by elevating SIRT1. However, the effect was not very apparent when we treated SIRT1^+/−^ mice with SRT2104 via intragastric administration (Figure S1). We believed that the inability to reduce knockdown-SIRT1-induced hypertension via intragastric administration was related to the drug dosage. SRT2104 used as 100 mg/kg/day for 24 weeks could significantly elevate SIRT1 protein in diabetic mice [24,25]. However, our intragastric administration could only use for 18 days during mice pregnancy. Our intraperitoneal results indicated that SRT2104 could be a new therapy for SIRT1 induced pre-eclampsia after more experiments on the safety in pregnant women.

P4 might improve the symptoms of pre-eclampsia. P4 is an essential hormone in the process of reproduction, and circulating P4 is significantly lower in PE women compared to controls [16]. There are pieces of evidence that early P4 treatment in ART improves the incidence and outcome of pre-eclampsia [50,51,52]. In addition, animal experiments also demonstrated that P4 could attenuate hypertension, improve inflammation, and boost fetal weight in a reduced uterine perfusion pressure rat model [16,17,53]. Our results also revealed that P4 could significantly reduce blood pressure and elevate fetal weight to prevent the aggravation of pre-eclampsia. Additionally, P4 treatment can promote trophoblast invasion and inhibit trophoblast apoptosis. This evidence demonstrated that P4 supplementation during pregnancy might play a role in the treatment and prevention of pre-eclampsia.

P4 might interact with SIRT1 to take effect in pre-eclampsia. It has been reported that SIRT1 deacetylated the hinge region of the P4 receptor to regulate the kinetics of PR nucleo-cytoplasmic transport and subsequent transcriptional activity [54,55]. In addition, knockdown of SIRT2, an isoform of the Sirtuins family, stimulated the mRNA abundance of CYP11 A1, which is a rate-limiting enzyme in P4 biosynthesis [56]. Inhibition of SIRT1 expression with nicotinamide (NAM) reduces P4 production in MA-10 cells [57]. This evidence showed that there is an interactive relationship between SIRT1 and P4. However, the direct mechanisms between SIRT1 and P4 in pre-eclampsia need to be further clarified.

Metformin can also reduce the blood pressure in SIRT1^+/−^ mice but causes fetal intrauterine growth restriction, which increases fetal morbidity and mortality. We concluded that this might be related to the weight-reducing effect of metformin [58]. However, several clinical research papers reported that metformin does not reduce the incidence of large gestational age newborns; it reduces the risk of neonatal intensive care unit admissions [58,59,60]. Related studies on metformin in the treatment of gestational diabetes and PCOS have shown that metformin can improve pregnancy outcomes [61,62,63,64]. However, these results are still controversial.

In our study, we innovatively confirmed that the SIRT1-knockdown mice showed typical pre-eclampsia-like symptoms, suggesting that SIRT1 is profoundly significant in the mechanisms of pre-eclampsia and might be a new therapy marker for pre-eclampsia. In addition, we found that P4 can significantly diminish the symptoms induced by SIRT1 knockdown. We believed that P4 presented a perfect effect on improving the symptoms of pre-eclampsia. However, there were some shortcomings in our research. We used whole-body SIRT1 knockdown mice rather than trophoblast-specific SIRT1 knockout mice. The SIRT1 knockdown is sufficient to present significant pre-eclampsia-like symptoms, and we are convinced that trophoblast-specific SIRT1 knockout mice might show more significant performances. In addition, the mechanisms of P4 in pre-eclampsia still need to be explored.

In conclusion, our study provides a new pre-eclampsia-like mouse model to explore the mechanisms of pre-eclampsia, mainly about SIRT1. In particular, we demonstrated that P4 could improve the symptoms of PE-like mice. Therefore, further studies should conduct basic experiments to conform the comprehensive mechanisms of SIRT1 in pre-eclampsia based on the mice model and examine relevant clinical evidence to indicate the effectiveness and potential side effects of P4 in the treatment of pre-eclampsia, which will be an innovative finding for the prevention and treatment of pre-eclampsia.

## 5. Conclusions

In the present study, we found that the expression of SIRT1 was relatively lower in the placentas and serum samples of pre-eclampsia patients. Additionally, SIRT1 knockdown (SIRT1^+/−^) mice showed typical pre-eclampsia-like symptoms, such as hypertension, proteinuria, fetal growth restriction, kidney injury, and a narrowed placental labyrinth layer. Interestingly, these performances could be improved after the intraperitoneal injection of SIRT1 agonist SRT2104. More importantly, we found that the efficacy of P4 on attenuating PE-like symptoms was profoundly better than that of metformin in SIRT1^+/−^ mice. In addition, our results suggested that P4 can promote the invasion and inhibit the apoptosis of trophoblasts. These data suggest that SIRT1 plays an important role in pre-eclampsia, and P4 alleviates pre-eclampsia-like symptoms mediated by SIRT1 deficiency.

## Figures and Tables

**Table 1:** Clinical characteristics of the study population.

	NP (*n* = 95)	PE (*n* = 76)	*p*-Value
Maternal age (year)	36.50 ± 2.774	38.00 ± 3.090	*p* < 0.05 *
Gestational age (week)	38.90 ± 1.447	38.75 ± 2.048	*p* > 0.05
Neonatal weight (g)	3263 ± 470.9	3204 ± 713.0	*p* > 0.05
Systolic BP (mmHg)	119.9 ± 9.835	136.4 ± 14.66	*p* < 0.0001 ****
Diastolic BP (mmHg)	73.89 ± 7.045	85.09 ± 11.79	*p* < 0.0001 ****
Proteinuria (g/24 h)	-	1.434 ± 2.515	-
FGR	4/95	14/76	*p* < 0.05 *

All data values are expressed as mean ± SEM. P values were obtained using the unpaired Student’s test on Graph Pad Prism Version 9.2.0. * *p* < 0.05; **** *p* < 0.0001. BP: blood pressure; NP: normal pregnancy (*n* = 95); PE: pre-eclampsia (*n* = 76); FGR: fetal growth restriction.

## Data Availability

Not applicable.

## Supplementary Materials

The following supporting information can be downloaded at: https://www.mdpi.com/article/10.3390/biom12030422/s1, Figure S1: SRT2104 for intragastric administration.
