# Supplementary material for: Progesterone Attenuates SIRT1-Deficiency-Mediated Pre-Eclampsia
Source: Biomolecules. 2022 Mar 9;12(3):422. doi: 10.3390/biom12030422 (PMC8946184; doi:10.3390/biom12030422)
Supplement: Supplementary file 1 [file biomolecules-12-00422-s001.zip › biomolecules-1565295-supplementary.pdf]

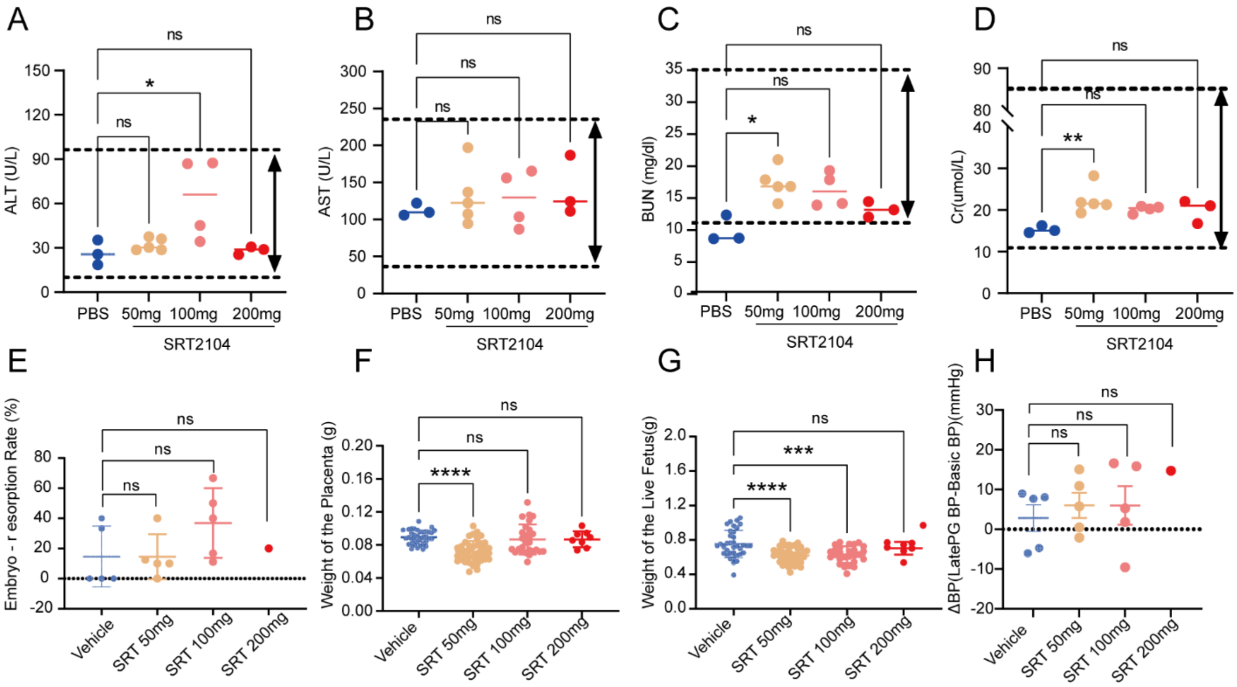

Figure S1. SRT2104 for intragastric administration. (**A**–**D**) The level of ALT (**A**), AST (**B**), BUN (**C**), and CR (**D**) of serum in mice with PBS ($n = 3$), SRT 50mg ($n = 5$), SRT 100mg ($n = 4$), and SRT 200 mg ($n = 3$), respectively. (**E**–**H**) The embryo-resorption rate (**E**), the weight of the placenta (**F**), the weight of the live fetus (**G**), and ΔBP (late PG—basic condition) (**H**) in mice with PBS ($n = 5$), SRT 50 mg ($n = 5$), SRT 100 mg ($n = 5$), and SRT 200 mg ($n = 1$), respectively. Error bars, mean ± SD. The data were analyzed by one-way ANOVA (and nonparametric or mixed) and unpaired Student's test (and nonparametric tests). * $P < 0.05$; ** $P < 0.01$; *** $P < 0.001$; **** $P < 0.0001$.
